# Supplementary material for: Effectiveness and Safety of Ayurvedic Medicines in Type 2 Diabetes Mellitus Management: A Systematic Review and Meta-Analysis
Source: Front Pharmacol. 2022 Jun 8;13:821810. doi: 10.3389/fphar.2022.821810 (PMC9213670; doi:10.3389/fphar.2022.821810)
Supplement: Supplementary file 1 [file DataSheet1.zip › Supplementary Material/Forest and Funnel Plots/Trigonella foenum-graecum/TG.pdf]

Trigonella foenum-graecum - TG

Study	Intervention N	Intervention Mean	Intervention SD	Comparator N	Comparator Mean	Comparator SD	Mean Diff. with 95% CI	Weight (%)
Gupta 2001	12	131.00	105.00	13	145.70	65.50	-14.70 [-82.70, 53.30]	7.22
Rafraf 2014	44	80.81	29.05	44	137.00	48.63	-56.19 [-72.93, -39.45]	19.86
Singh 2016	20	159.20	25.89	20	198.47	33.36	-39.27 [-57.78, -20.76]	19.37
Gholaman 2018	10	155.46	13.03	10	161.69	9.60	-6.23 [-16.26, 3.80]	21.40
Kandhare 2018	48	187.35	125.50	53	162.62	77.96	24.73 [-15.61, 65.07]	12.87
Rashid 2019	32	166.38	46.91	32	194.70	27.44	-28.32 [-47.15, -9.49]	19.28
**Overall**							-23.44 [-45.64, -1.23]	

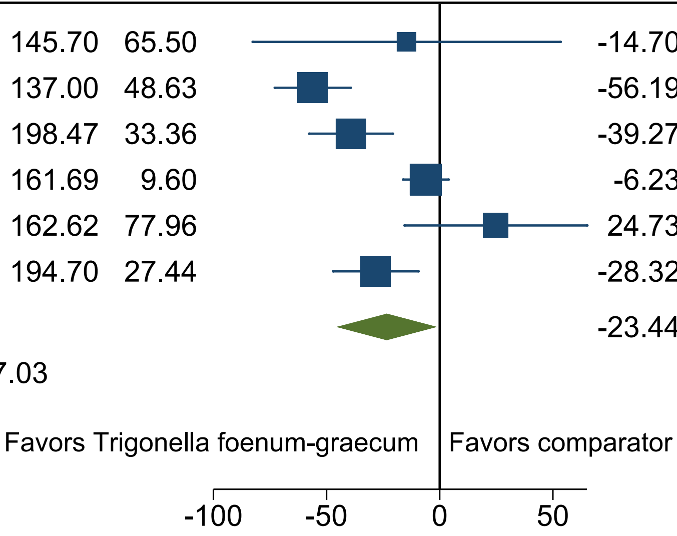

Heterogeneity: $\tau^2 = 573.21$, $I^2 = 85.77\%$, $H^2 = 7.03$

Test of $\theta_i = \theta_j$: $Q(5) = 34.50$, $p = 0.00$

Test of $\theta = 0$: $z = -2.07$, $p = 0.04$

Random-effects REML model
